# GPS-Based Hidden Markov Models to Document Pastoral Mobility in the Sahel

**DOI:** 10.3390/s24216964

**Published:** 2024-10-30

**Authors:** Arthur Scriban, Serge Nabeneza, Daniel Cornelis, Etienne Delay, Jonathan Vayssières, Jean-Daniel Cesaro, Paulo Salgado

**Affiliations:** 1 CIRAD, UMR SELMET, PPZS, Dakar 11500, Senegal; paulo.salgado@cirad.fr; 2 CIRAD, UMR SELMET, F-34398 Montpellier, France; serge.nabeneza@cirad.fr (S.N.); jean-daniel.cesaro@cirad.fr (J.-D.C.); 3 SELMET, Université de Montpellier, CIRAD, INRAE, Institut Agro, F-34398 Montpellier, France; 4 CIRAD, UPR Forêts et Sociétés, F-34398 Montpellier, France; daniel.cornelis@cirad.fr; 5 Forêts et Sociétés, Université de Montpellier, CIRAD, F-34090 Montpellier, France; 6 CIRAD, UMR SENS, Dakar 10700, Senegal; etienne.delay@cirad.fr; 7 UCAD, École Supérieure Polytechnique de Dakar, Dakar 10700, Senegal; 8 UMMISCO, Université Cheikh Anta Diop, Dakar 10700, Senegal; 9 CIRAD, UMR SELMET, F-97410 Saint-Pierre, La Réunion, France; jonathan.vayssieres@cirad.fr

**Keywords:** herding practices, agropastoral systems, transhumance, cattle behaviour model, GPS collar, semi-arid rangeland, West Africa

## Abstract

In agrarian systems where animal mobility is crucial for feed management, nutrient cycles and household economy, there is a notable lack of precise data on livestock mobility and herding practices. We introduce a methodology leveraging GPS-based behavioural models to analyse and document pastoral mobility in the Sahel. Over 2.5 years, we conducted a continuous collection of GPS data from transhumant and resident cattle herds in the Senegalese agropastoral semiarid rangelands. We developed a Hidden Markov Model robustly fitted to these data to classify recordings into three states of activity: resting (47% overall), foraging (37%) and travelling (16%). We detail our process for selecting the states and testing data subsets to guide future similar endeavours. The model describes state changes and how temperature affects them. By combining the resulting dataset with satellite-based land-use data, we show the distribution of activities across landscapes and seasons and within a day. We accurately reproduced key aspects of cattle mobility and characterised rarely documented features of Sahel agropastoral practices, such as transhumance phases, nocturnal grazing and in-field rainy season paddocking. These results suggest that our methodology, which we make available, could be valuable in addressing issues related to the future of Sahelian pastoralism.

## Simple Summary

We propose a behaviour model for West-African pastoral mobility based on a long-term survey of agropastoral cattle herds in Senegal. We combined position and activity statistics with satellite land-use data. Our findings on behaviour and herding practices align with literature and field observations and shed light on nocturnal foraging, transhumance phases and individual fallowing. We describe and discuss our methodology to provide tools for future research.

## 1. Introduction

The African Sahel region has a strong climatic seasonality. Rainfall is scarce and concentrated to a restricted rainy season, occuring during the summer months of the Northern Hemisphere. For this reason, vegetation growth is limited and highly seasonal. Forage availability on the margins of the Sahara only occurs at the height of the rainy season, while, at this time, crops are grown in the southern part of the Sahel. Pastoral livestock systems are organised around these biogeographic constraints. Herds generally travel long distances from south to north and back, twice a year, to follow vegetation growth—a practice known as transhumance. Additionally, livestock breeding in this region is a highly multifunctional activity. It provides income to rural households, serves as a capital reserve and has strong social significance, among other things [1]. It is also the principal means of fertility transfer, via manure, in an environment where sandy soils require sustained inputs to ensure productivity [2]. Although not all livestock systems are mobile, the most common form of cattle farming consists of herds moving relatively freely during the long dry season, feeding on rangelands and crop residues left on the field after harvest [3]. At night, the cattle are paddocked in barren, cultivated parcels to ensure the return of manure to these soils. These practices result in mixed agricultural systems, in which livestock systems are integrated with crops while remaining extensive. Transhumance and the combination of free grazing and night paddocking make pastoral livestock farming essentially mobile.

Although the value of pastoral mobility in the Sahel is well recognized [4], there is a distinct lack of high spatial resolution and long-term data to document it. Such data can be highly valuable for analysing herding decisions over years and seasons, documenting how cattle move across the landscapes or for precisely characterizing the feeding habits of animals and documenting the quantity and quality of their intake over time [5]. The aim of this paper is to present methods to make the most of GPS surveys to understand agropastoral livestock practices, using agropastoral systems of Senegal as a case-study, both with and without transhumance. We consider mobility as a combination of herd movements and the factors determining them, employing a behavioural model as the foundation of our analysis, with inspiration from methods commonly used in the field of ecology.

**Figure 1:**
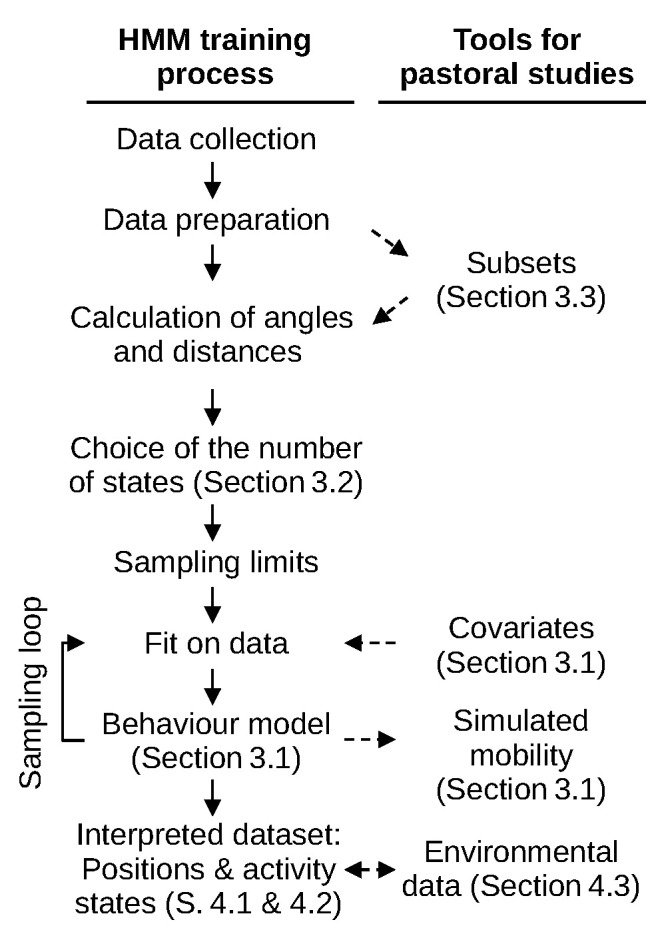
Behaviour model training process and useful tools illustrated by results of the paper.

This paper begins with a brief introduction to cattle mobility in this case study. It is followed by a description of our survey protocol, the data preparation routine we developed (summarised in Figure 1) and how we trained a behavioural and statistical Hidden Markov Model (HMM) on the collected data. We then present the resulting model and a few additional results from the training process which provided us with valuable methodological insights. The next section puts forward thematic results from activity data analysis, which we combined with spatial land-use data from satellite imagery. Then, we discuss the limits of our approach, the validity of our model and how our empirical results compare with literature and field observations. We highlight methodological features that may be of use to pastoralists and conclude by proposing several thematic issues that our approach may help address. Both our dataset [6] and data processing routine [7] are made openly available.

## 2. Materials and Methods

### 2.1. Case Study: Senegal Pastoral Livestock Systems

Senegal is the westernmost country in the Sahel region. Its climate is highly seasonal, with a rainy season occuring from June to the end of October in our study area, which accounts for most, if not all, of the annual rainfall. Our study area intersects two climatic areas. We have divided it into two parts: the agropastoral region to the South and the pastoral area to the North. The southern part is in the warm and semi-arid Sudanian climatic zone (Aw in the Köppen classification; see Kottek et al. [8], for example). Average rainfall is 551 mm/year [9], concentrated exclusively between June and October. Our study area extends from the area surrounding the town of Mbour, at the West of the old groundnut basin (14°20′ N, 16°40′ W), up to the vicinity of Tambacounda to the East (13°50′ N, 13°50′ W), where annual rainfall is 582 mm/y [10]. The farmers whose herds we followed live in the westernmost part of this area, in what we refer to here as the home villages, or villages of origin. The northern part of our study area is in the pastoral region of Ferlo, where the Sahelo-Sudanian climate (BSh) corresponds to lower annual rainfall, about 263 mm/year [ibid.], which starts later and ends earlier in the year.

In the southern part, groundnut and millet are cultivated in biannual successions under tree cover with a variable density of various species, some of which are used as a source of fodder. The landscape is divided into units exploited by the villages, organised concentrically around dwellings, with a continuous area of cultivated parcels around them and a patchwork of grazed rangelands and wet lowlands at the outer boundaries (as described by [11]). In the northern pastoral part, the landscape is essentially savannah, and the anthropogenic use of vegetal biomass mainly consists of feeding livestock [12].

Livestock farming practices in the old groundnut basin are strongly constrained by the seasonality of rainfall. During the rainy season, crop growth requires livestock to be kept away from cultivated parcels. Several strategies are used for this purpose [13], two of which involve cattle herd mobility and are considered in this paper. The first strategy is to cut off their access to the cultivated parcels by the systematic guidance of herders, most often children of the family, and secondly by blocking access with barriers made from stacked thorny branches. Animals are only allowed in the lowlands and rangelands on the edge of the villages. The disadvantage of this practice is that space and biomass are limited [11]. In some specific cases, this strategy may be supplemented by collective fallow. Each year, farmers collectively decide to leave a group of adjacent parcels uncultivated to allow spontaneous vegetation to grow, where cattle are restrained, day and night, by barriers of thorny branches throughout the rainy season. The fallow area is connected to lowlands and rangelands [14]. In practice, rangelands are scarce, laying fallow is a rare legacy practice and most farmers send their herd on transhumance [15], the second strategy. Transhumance requires a set of conditions to be met, in terms of herd size, human and material capital and social network [16,17,18]. It involves the gathering together of droves from one or several herds and setting off for the pastoral area. Households or families can send their own drove or combine herds and resources with others. Droves return at the start of the dry season, when forage dwindles in the north.

During the dry season, when crops are harvested and residues are left accessible on the ground, croplands are open to free-grazing animals, including cattle. During the day, they are herded but allowed relatively free movement to search for feed on their own. At night, the herds are paddocked, either tethered to posts or confined within barbed wire fences. Their owners choose their location and carry out a rotation every 3 to 4 days on parcels to be cultivated during the rainy season to benefit from fertility transfers.

### 2.2. Sampling Design, GPS Collars and Data Collection

The survey was carried out on herds belonging to farmers located in three villages: Barry Sine, Diohine and Sob. In these villages, a variable proportion of cattle herds leave the village for transhumance during the rainy season, while the rest are kept within the village area. In Diohine, farmers additionally engage in traditional collective fallow practices. These villages were chosen to be representative of the livestock management systems of the agropastoral region. Farmers were chosen under the guidance of village leaders, with the objective to cover the two mobility strategies for the rainy season described above.

At the end of the 2021 dry season (May), adult cows belonging to separate farmers were fitted with 15 GPS collars. Zebu cows of the Gobra breed (see [19]) were selected by their owners above all for their strong character to minimize the risk of theft. We considered the movement of a GPS-tracked cow to be representative of the entire herd, on the basis of our field observations and our own knowledge of herd management. GPS collars (Vectronic *Vertex Lite* collars; *Vectronic Aerospace GmBH*, https://www.vectronic-aerospace.com/vertex-lite-collar/, accessed on 4 July 2024) were programmed to record a location every 30 min, with an advertised accuracy of around 95% within a 10 m radius. The embedded thermometer, which records the temperature of the air around the animal, was set to record its readings at each GPS acquisition. The expected lifespan of the lithium-ion batteries installed in the collars at this rate has been estimated at approximately 2 years. Data from the collars were downloaded in the field every three to four months using a USB receiver and Vectronic’s *GPS Plus X* software on a standard computer. For this, the herds were located in advance using a subset of location data sent via Iridium mobile communication (three GPS locations per day, sent every three days), which were not used in further calculations.

The survey continued until December, 2023, for a total of approximately 31 months, or two full years plus an additional rainy season and part of a cold dry season. During the study, a total of 21 individual cows from 19 separate herds were monitored. For various reasons, e.g., the death or sale of the animal or on the decision of its owner, some collars were shifted between animals during the study, from the same herd whenever possible. Although our initial objective was to sample equal numbers of transhumant and resident herds, results showed that 12 of the 19 herds undertook transhumance during the rainy season, sometimes contrary to the initial intention of their owner. It should be noted that a few GPS collars ended up malfunctioning partially or completely.

### 2.3. Preparation of GPS and Satellite-Based Land-Use Data

GPS data were prepared and cleaned using the Herds Activity Mapping and Analytical Classification (HAMAC) routine, developed for the purpose of this study. The complete and reproducible code is available at https://gitlab.cirad.fr/selmet/hamac (accessed on 15 October 2024) [7]. The HAMAC routine is written in the R statistical programming language (v. 4.3.3) [20], relies on the *moveHMM* package (v. 1.9) [21], for several key features and makes extensive use of parallel computing for data processing. The HAMAC routine includes tools to wrangle, clean and format data, prepare and combine data from multiple sources, train and explore HMMs and generate diverse figures and metrics on the model and final dataset. It requires two datasets. Firstly, a table of time-stamped GPS readings with the collar ID, latitude, longitude, the GPS sensor dilution of precision (DOP; not strictly required) and the recorded temperature is required. Secondly, a metadata table, containing the periods during which each animal was fitted with each collar and information about the herd and the farmer is required.

During the study, 573 591 GPS locations were collected, with an average DOP of 1.188. After filtering out incomplete data and sequences with inconsistent observation frequencies, including valid individual observations included in unusable sequences to avoid data gaps, the usable data comprised 560 490 GPS points, resulting in approximately 2.3% of the data being filtered out. Additional attribute data were then assigned to the GPS location dataset. Seasonal and diurnal variables were added based on time and geographic position relative to sunrise and sunset at the geographical centre of the dataset. Euclidean distances and relative angles between successive uninterrupted locations for each GPS collar were also calculated using the *moveHMM* software package.

To combine location and activity data into geographical information, we used a couple of land-use maps. The first covers a 450 km^2^ area around the home villages and consists of a land-use map derived from a 1.5 m resolution multispectral SPOT7 satellite scene for 6 January 2016, built up by Ndiaye et al. [22], with a maximum likelihood algorithm. The second covers the entirety of Senegal and thus the whole study area. The original image, a Globcover product from 2005 [23], is of a massively lower resolution (300 m) than the SPOT one. We harmonised land-use classifications for both maps using a simplification of the standard FAO/UNEP Land Cover Classification System legend [ibid.]. We used the QGIS software [24] to associate each GPS observation with the vector shape of each land unit using the *join attributes by location* function and spatial indexes to optimise the process.

### 2.4. The Hidden Markov Behaviour Model

Identifying the interaction between a herd and its direct environment with pure positional information would prove challenging. We relied on the conceptual framework developed by Nathan et al. [25], which relies on the postulate that animal mobility behaviour and herding can be discretised into a set of finite states. The herd is considered a system, being in a single state of mobility at a given moment and changing or remaining in that state under different conditions over time. The activity model we propose in this paper was determined to simply differentiate three states in its simplest form, which we identified to be Resting, Foraging and Travelling. In the resting state, herds have little or no mobility. The foraging state encompasses grazing or browsing on plant biomass, as well as the research movements made to find suitable vegetation patches to do so. In the travelling state, the herd was driven over long distances. As discussed in Section 3.2, this number of three states was chosen to be large enough to ensure that the state which involves biomass intake emerges, while a higher number of states would have hindered interpretation and generalizability and would have required heavy additional computation. States names vary in the literature for cattle mobility models (e.g., [26,27,28,29]). We believe that the names we chose represent more accurately the behaviour of free-grazing agropastoral herds at our time resolution.

We considered that for each 30 min step, i.e., each “displacement between two successive positional records of the organism” [25], the behaviour of the herd could be mainly attributed to one state rather than to another. Thus, each herd was modelled as being in just one of these activity states for the duration between each GPS observation, similar to a finite state machine. We considered as a hypothesis that the transition from one observed state (over a given period) to another had a fixed probability, which only depended on the state taken by the system in the previous period. This corresponds to the Markov model paradigm. A 3×3 matrix Γ of conditional probabilities could thus be emitted as a “behaviour” model by analogy with ethology, which, in the case of Sahel cattle herds, corresponds partly to spontaneous animal behaviour and partly to human-driven herding decisions. As a refinement of the model, the state transition probabilities in the Γ matrix could, in the process of fitting the model to the data, depend linearly on one or several exogenous covariates. The scalars relating these covariates and the conditional probabilities illustrate how state transitions varied as a function of these variables over the acquisition period. We attempted this with collar-measured temperatures as a proof of concept but did not include covariates further into this study.

Since activity states are latent, i.e., the behaviour is not explicitly discernable in the GPS position data, the model is a hidden Markov model (HMM). We required an observable proxy to infer the state of the herd for each observation. We used the Euclidean distance between two points (step length) and the angle between two successive observed trajectories, which we called trajectory metrics. Evidently, the observed trajectories differed from the path actually taken by the animals constituting the herd, but these could nevertheless be considered the expression of the underlying activity states. Each theoretical state *i* was associated with a pair of statistical distributions for the emission of a pair of trajectory metrics. Step lengths followed normal distributions of means $\mu_{i}$ and standard deviations $\sigma_{i}$ in km. For the angles, we used Von Mises distributions (normal law on the circle), each with a mean $\mu_{i}^{\prime }$ and a concentration $\kappa_{i}$ in radians. To determine the matrix Γ of the Markov model most likely to have emitted the observed sequence, we used a recursive statistical inference algorithm (nonlinear minimisation; NLM) integrated into the *moveHMM* package [21]. This algorithm judges the models it produces by their likelihood relative to the set of observed GPS data. Likelihood (L) is a metric that expresses the probability of observing a set of observations, assuming that the model in question produced it, hence its name. The NLM algorithm starts with an initial set of parameters ${\left(\mu_{i},\;\sigma_{i},\;\mu_{i}^{\prime },\;\kappa_{i}\right)}_{i\in [\;[1,3]\;]}$, iteratively generates new models and computes their likelihood, up to the point where it is maximal (in reality, where −log(L) is minimal). The model it stabilises on is thus the most likely given the dataset.

As NLM is purely heuristic, similar to a hill-climbing algorithm, the initial set of parameters it is given greatly influences the path it may take and may lead to a local optimum. Therefore, it was necessary to sample the parameter space to find a global optimal model. We performed direct sampling of 110 initial parameter sets (for a reasonable computation time on the 22-core machine at our disposal) with uniform distributions for each parameter, between the values given in Table 1. These values were determined from an initial observation of distance and angle distribution within the entire dataset and expert knowledge on pastoral mobility. Each sample produces a most-likely model, and the one with the highest likelihood of the batch, which we call the optimal model, is selected.

Once the optimal model was determined, the Viterbi algorithm (implemented in the *moveHMM* package) was used to associate each observation with its most probable activity state.

## 3. Methodological Results—Producing an HMM for West African Pastoral Systems

### 3.1. Activity States Definition and Markov Behavioural Model

The direct sampling resulted in 87% of the fitting replications leading to a single optimal model, with a log-likelihood of −197,099, which is an improvement of 71% compared to the null hypothesis of a single-state model. The fitting process for this model took a number of iterations varying from 147 to 342, depending on the starting point, with an average of 226. It is noteworthy that 20% of the sample of iterative processes, which produced the optimal model, had a reversal of the foraging and travelling states relative to the initial parameter set, which does not affect their validity nor indeed change the value of their likelihood. The rest of the replications, with one exception, led to a single, locally optimal model, with a log-likelihood of −240,810. The computation time on a 22-core machine was 65.5 h.

The parameters of the distributions of emission of the trajectory metrics associated with each of the three states of the optimal model are presented in Table 1 and represented in Section 3.3. There is a clear distinction between the states, with significantly different and narrow confidence intervals. The resting state is characterised by very short step lengths and angles centered on −π (i.e., π as well). Although seemingly confusing at first, this last point is common and is due to distances between locations being very close to the precision of GPS receivers (e.g., [21]). The model associates an observation with the foraging state for a step length of approximately 180.1 m, i.e., speeds around 0.360 km/h and angles centered on 0 rad but fairly uniformly distributed across the circle. This corresponds to a research pattern in which cattle look for resources to browse or graze. The travelling state was associated with long (1.37 km/h), reasonably straight trajectories (angles highly concentrated around 0) in which the herds move towards a predetermined destination. The last row of Table 1 shows as an illustration a movement simulation for the three states carried out with the *simData* function of the *moveHMM* package.

**Figure 2:**
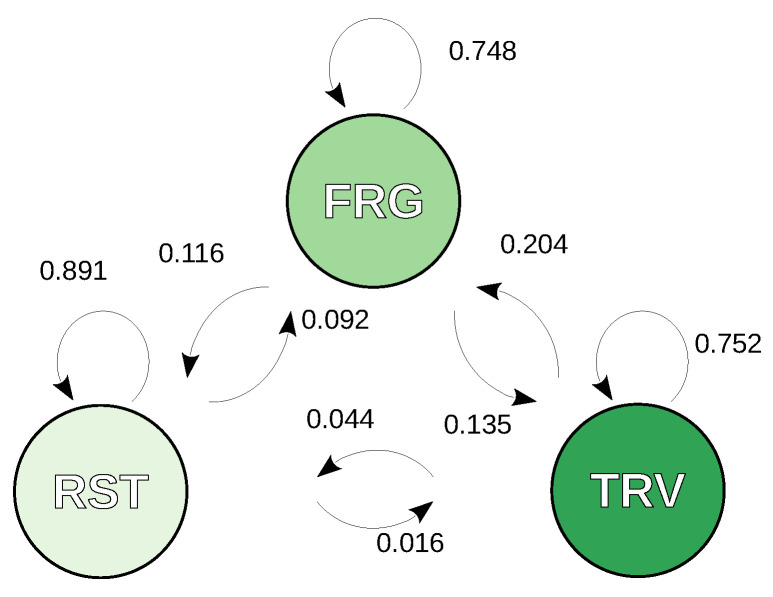
Three states behavioural model and conditional probabilities to switch or remain in a state every 30 min step, given the last state. RST: resting, FRG: foraging, TVL: travelling.

Figure 2 is a schematic representation of the Γ matrix of transition probabilities between states for each time step. All states are accessible from one another and fairly stable, with about a three in four chance every 30 min of remaining in foraging and travelling states and almost a 90% chance of resting to last, reflecting relatively long streaks of the rest state in the dataset. The transition from resting to foraging (research and intake) is significantly more likely than the transition to travelling. Leaving a travelling state, on the other hand, leads to foraging five times more often than to resting. Leaving a foraging state can lead equally to resting or travelling. The initial-state distribution matrix, of no relevance to our discussion but necessary to reuse the model, is the following: Π=[0.41580.32930.2549], in the order: resting, foraging, travelling.

**Figure 3:**
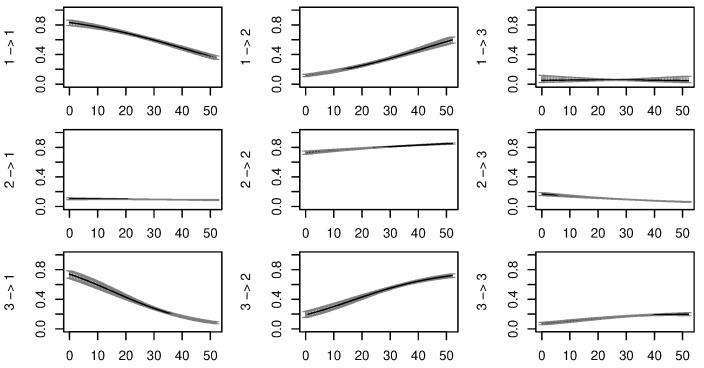
Transition probabilities between states as a function of the temperature recorded by the collars (°C) and confidence intervals. 1: resting, 2: foraging, 3: travelling.

Figure 3 shows how these transition probabilities varied with temperature as a covariate over the duration of the survey. The actual range of measured temperatures, shown in the Supplementary Figure S1, rarely exceeds 45 °C or drops below 25 °C. The transition probabilities beyond these points in Figure 3 should therefore be considered extrapolations. The resting state loses stability and increasingly leads to a foraging behaviour as temperatures increase. Likewise, leaving the travelling state when temperatures are higher will more likely mean slowing down to reach a foraging state than settling to rest. The stability of the foraging state appears mostly unaffected by the temperature.

### 3.2. Selecting the Number of States

To determine the appropriate number of states for the HMM in response to our research question, we adopted a heuristic approach, inspired by Pohle et al. [30]. We trained a set of models on the dataset with an increasing number of behavioural states while observing the evolution in likelihood. Naturally, adding states improves the likelihood, as the model has more flexibility to fit the data. Therefore, we adopted an Occam’s razor approach, seeking, in addition to an improvement in the goodness of fit, the minimum number of states that allow the emergence of a grazing state, as indicated in Section 2.4. The metric we use for this purpose is the Bayesian information criteria, BIC=kln(n)−2ln(L), with *k*, the number of parameters of a model and *n*, the number of observations. The BIC allows to compare the quality of the adjustment by taking into account the increase in the complexity of the models (here, *k* is the number of states). The parameter limits for sampling are similar to those shown in Table 1, and the results are presented in Table 2.

With a two-state model, we obtained a first state with short distances and relatively random angles, centered on approximately −π, which is similar to what we described in Section 3.1 and appeared to be a state of rest. The second state has straighter trajectories with longer steps, which largely covered any movement of the herd. There is, in fact, no way to assess with certainty whether or not an observation in state 2 involves vegetation intake or not. The three-state model is described above, in Section 3.1, and a foraging state can be clearly identified. States 3 and 4 of the four-state model are very similar to the foraging and travelling states of the three-state model. It is the resting state which appears to have been split between a “true resting” state, in which the collar remains almost motionless (below the precision of the GPS sensors), and an “active resting” state, in which a certain distance is travelled in relatively random directions (low concentration κ). The five-state model is very similar to the latter for its first three states and distinguishes two substates of the travelling state. These additional states do not produce a significant increase in fit, which corresponds to fitting to noise in the distribution of the data. Thus, they are of little interest when discussing West African cattle grazing and come at the cost of increased computation time. For these reasons, we have decided to keep the three-state model in this study. Nevertheless, these additional states may be of interest for more in-depth studies on different aspects of cattle herding and behaviour.

### 3.3. Fitting Models on Subsets of Data for Transhumant and Resident Herds

**Figure 4:**
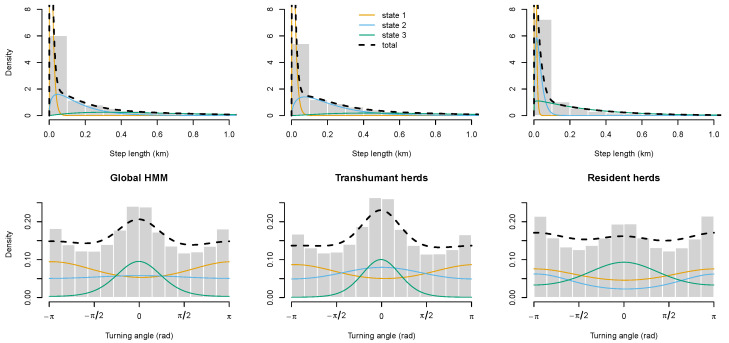
Distributions of trajectories length (grey histogram top) and angles (bottom), with density of probabilities for their emission for each state and the total, for the whole dataset (global hidden Markov model–HMM; left), transhumant herds (middle) and resident herds (right). Step length abscissa (top) is artificially truncated to 1 km for readability purposes.

We had as an initial hypothesis that a unified behavioural model would accurately distinguish states for both transhumant and resident herds. To confirm this, we carried out some light sampling (44 runs each, with the same protocol as that described in Section 2.4) on both parts of the dataset (352,125 observations for the 12 transhumant herds, 192,579 for the 7 resident herds). Neither the likelihood nor the BIC of the optimal models ajusted on each subset can be used to compare fit quality, as datasets differ and the design of experiment does not allow to quantify the validity of dividing the dataset. Nevertheless, trajectory emission parameters can serve as empirical evidence of broad similarity or dissimilarity. Figure 4 shows, for the whole dataset and each subset, the density of the probability laws of emission of a pair of length and angle variables for each of the three states. The HMM adjusted on transhumant herds only (middle) is very similar to the model on the whole dataset (left), and the three states found by the fitting process are those we discussed in Section 3.1: resting, foraging and travelling. The resting state is nearly identical (μ=14.3m,σ=12.7m,μ’=−3.0rad,κ=0.27rad). While the foraging and travelling states display slightly longer steps, with a marginally higher concentration of angles (foraging: μ=205m,σ=167m,μ’=0.013rad,κ=0.24rad; travelling: μ=721m,σ=458m,μ’=−0.020rad,κ=2.3rad). On the other hand, the optimal model trained on resident herds ends up with a rather different set of states. The first state is similar to the resting state of the global model (μ=7.25m,σ=5.4m,μ’ = −3.1 rad, κ=0.25rad) and the third to its foraging state ($\mu =290\;m,\;\sigma =280\;m,\;\mu ’=-1.5\cdot 10^{-4}\;\mathrm{rad},\;\kappa =0.52\;\mathrm{rad}$). However, instead of isolating a travelling state, as in the overall model, this dataset yields what appears to be a sort of “active resting” state, similar to that discussed in Section 3.2. The distances are short and angles very random, with the average around −π/π, which corresponds to little or no movement (μ=31.4m,σ=22.5m,μ’=−3.1rad,κ=0.50rad).

To sum things up, the model adjusted on the entire dataset is very similar to a model based on data from exclusively transhumant herds. On the contrary, a model fit on resident herds would require at least four states to isolate a foraging state.

## 4. Thematic Results—Using the Observed Trajectories and Their Associated Activity States

### 4.1. Identifying Key Features of Herd Behaviour

The Viterbi algorithm, applied to the sequence of hidden states using the optimal model, provides the following distribution of states for the entire dataset: resting 47%; foraging 37%; travelling 16%. Table 3 shows that on average, a resident herd spends 30% of the daytime period foraging, while it accounts for 39% of a transhumant herd’s day. Total distance travelled per day is also higher. Transhumant herds thus appear to have been more active than their resident counterpart during the survey.

**Figure 5:**
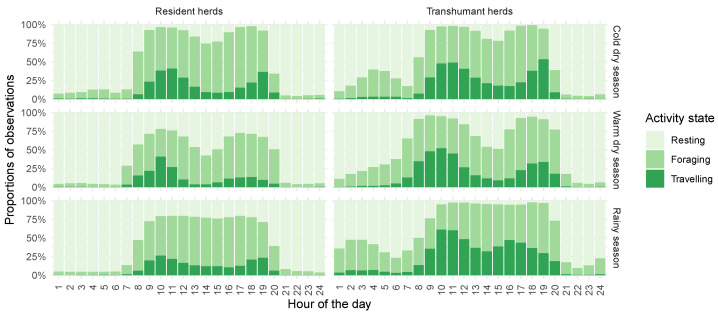
Temporal distribution of activity states by hour of the day, categorized by herd type and season. Cold dry season: November–February, warm dry season: March–May, rainy season: June–October.

Figure 5 presents the cattle activities recorded throughout the survey per hour for each season. A clear distinction is visible between night and day, with the resting state predominant at night and foraging and travelling prevailing during the day. The length of the active daytime period varies with the seasons, reflecting changes in the duration of daylight.

From the rainy season to the warm dry season, herds shift their movement patterns from grazing both morning and afternoon to predominantly grazing in the morning, taking advantage of the cooler temperatures. Movement is reduced during the rainy season due to the availability of resources in proximity, increases in the cold dry season as resources become more dispersed and decreases again in the warm dry season to conserve energy. Additionally, there is a noticeable decline in nighttime activity from the rainy season to the warm dry season.

Diurnal foraging occurs at a fairly constant proportion of points per day. Notably, rest peaks, discussed below, induce a decrease in travelling propensity, while foraging does not appear to decrease in proportion of time points. Additionally, nocturnal foraging by transhumant herds seems to compensate for the toll that travelling takes on daytime foraging, preventing them from meeting their feed capacity during the day. Foraging thus appears to require a fixed amount of time per day, which cannot be reduced.

Daytime rest periods are visible. In the rainy season, they are distributed evenly throughout the day for both types of herds, although they are rare for transhumant herds. Resident herds appear to rest about 25% of the time, at any time of the day. During both parts of the dry season, a clear peak in resting states is noticeable between noon and 3 p.m. in all cases. It is more pronounced during the warm dry season, when mid-day temperatures peak and forage availability is low, reaching about 50% of points at its peak for both herd types. This occurs when the transhumant herds are in their home area. This seasonal effect appears to be compensated by an increase in time spent foraging and travelling in the morning and the afternoon.

Resident herds, tethered during the night, are observed to be almost entirely inactive. The nocturnal activity of transhumant herds stands out. A fair amount of night foraging occurs during all seasons, mostly during the last half of the night. It can occasionally be associated with travelling, mainly during the rainy season, during which these herds are in the pastoral zone, which tends to suggest extensive night foraging in areas away from the drovers’ camp. Nocturnal foraging is more surprising in the dry season, as transhumant herds are in the agropastoral zone, either in their village of origin or in transit. This may be a demonstration of some strength of habit for animals used to open pastoral landscapes and means that they are not always tied up during the night.

### 4.2. Transhumance as Phasic, Seasonal Journeys

**Figure 6:**
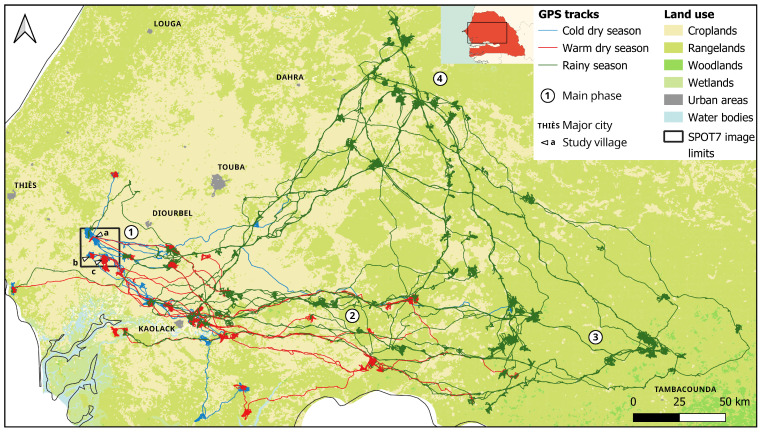
Full (31 months) GPS tracks for 14 transhumant herds, coloured by season, geographic extent of the SPOT land cover dataset (1.5 m resolution) and location of the home villages and main transhumant phases, displayed over land-use data from the Globcover dataset (300 m; WGS84). Home villages: a: Barry Sine, b: Diohine, c: Sob; phase numbers are referred to in the text.

Figure 6 shows the extent of the traces of the 12 transhumant herds across Senegal coloured according to each season. It is relatively clear that the transhumance movement is phasic.

The droves leave the home area (1) and first head east. Departures take place between March and the end of June (mean: mid-May, SD: 0.9 months). This coincides with the tilling periods, and earlier departures may be due to low forage availability. The first part of the journey (2), during the warm dry season, takes place in the western part of the former groundnut basin. It is an agropastoral area, where the landscape is similar to that of the villages of origin but with a less dense population and more prevalent rangelands. In periods of this phase when movement occurs, droves walk for a few days in a direct manner, while stopping at night, towards a location where they settle temporarily for several days or weeks. The duration of these stops varies considerably, and three clusters appear in the data: one with stops lasting an average of 11 days, another where stops last around 30 days and the last with the longest stops being measured at over two months. The start of the rainy season in the agropastoral area does not directly lead to movement towards the north, and the droves continue to move eastward, into areas mainly composed of uncultivated rangelands (3). A new phase of direct movement northward, toward the pastoral area, occurs in August, when forage become sufficiently abundant in the Ferlo zone (see [31] on the evolution of the seasonal front). The “pastoral” phase, in the Ferlo area (4), during the rainy season, lasts an average of 88.3 days (SD = 45.6 days), until the beginning of October. Discussions with the farmers surveyed suggest that departures from the pastoral zone correspond to the end of the harvest period in the agropastoral zone, when free grazing can resume without risk of damaging crops and when residues are made available. Biomass in pastoral areas is also under strain at that time. One may note that some herds appear to take the same path every year and that several herds appear to travel alongside one another.

### 4.3. Spatial Repartition of Cattle Activities

**Figure 7:**
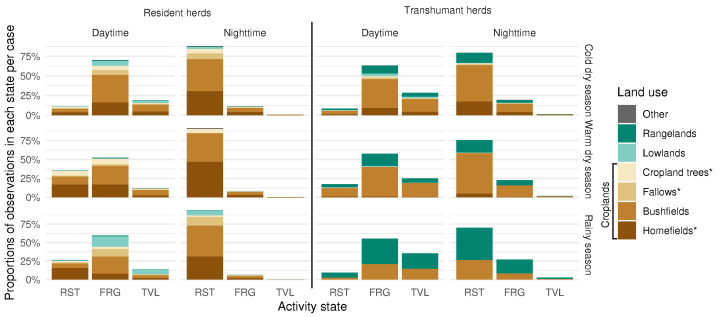
Repartition of GPS fixes per activity state coloured by landscape unit, for each season, herd type and for day and night. RST: resting, FRG: foraging, TVL: travelling; cold dry season: November–February, warm dry season: March–May, rainy season: June–October. Daytime and nighttime relative to sunrise and sunset each day. Water bodies are included into lowlands, dwelling areas are included into homefields, other land uses cover gardens and roads. Cropland units with an asterisk are a feature exclusive to the home area map, and the Globcover dataset encompasses them under the unit bushfields.

Figure 7 shows which landscape unit, as interpreted from satellite imagery, each GPS observation was made in, sorted by activity state and with a distinction between residents and transhumants between seasons and between night and day. Cropland trees are trees identified as such in the cropland areas of the SPOT7 image (see S2).

Regarding resident herds, daytime foraging and (tethered) nighttime resting occur primarily in cultivated areas (croplands) in all seasons. The majority of points were recorded in bushfields, which represent the largest areas. As the dry season progresses and crop residues become scarcer, homefields appear to become increasingly frequented. This is perhaps because hay and millet straw, stored around dwellings, are given to cattle to bridge the forage gap. During the rainy season, foraging and travelling occur more frequently in lowlands and fallows, where spontaneous vegetation is expected to be relatively abundant, although areas marked as cultivated remain the majority. Cultivated parcels are supposed to be fenced and inaccessible to cattle, and almost no activity in these areas was expected, apart from the village where collective fallowing takes place. However, we show that not only do cattle graze more in these areas than in lowland and rangelands but that herds are also stationed at night in cultivated parcels, which is confirmed by direct observation of the spatialised data. This suggests a form of individual fallowing on the part of farmers who do not send their herd on transhumance for various reasons. Aside from this, daytime rest appears to occur wherever foraging occurs, as the proportions between land units are roughly the same between both activities. During the dry season, resident herds spend 31% of the total daytime within homefields, mostly foraging, and 45% during nighttime, almost exclusively resting. It is thus reasonable to assume that fertility transfers occur towards homefields.

Transhumant herds, during the cold dry season, reside in the villages of origin and are herded in the same way as the resident herds. Indeed, the grazed landscapes are approximately the same as those of the latter, with, however, a greater proportion of rangeland and bushfields. These land units are generally further away from concentrated dwellings, where water wells the animals drink at and parcels chosen for night corralling are generally located. This may explain the longer time spent travelling during this season (as shown in Section 4.1). Part of these data can also be explained by anecdotal individual behaviour, as may be inferred from tracks (Figure 6). Coarser landscape data are used outside the original village area (see Figure 6). In particular, homefields and bushfields have not been differentiated and are grouped under the umbrella category of bushfields. Thus, the interpretation of the results shown on the middle and bottom left parts of Figure 7 should be made with caution. During the warm dry season, as discussed in Section 4.2, most of the droves formed and left for the first part of the transhumance, in the eastern agropastoral zone (zone 2 in Figure 6). In this region, foraging states were recorded in cultivated areas and, to a lesser extent, in rangelands. Nighttime resting seems to occur in cultivated areas, but more precise analysis is needed to determine whether or not manure contracts are made between herds and landowners. For both parts of the dry season, nocturnal foraging and travelling identified in Section 4.1 occur in the same landscape units as during the day. Foraging during the wet season is mainly recorded in rangelands, and it is possible that, given the low resolution of the land-use dataset, areas labelled as “bushfields” in that part of the country (4) are actually grasslands. As illustrated in Figure 6, the first part of the rainy season occurs, for most herds, in the agropastoral zone (3), and fertility transfers can take place.

## 5. Discussion

### 5.1. Limits Inherent to the Data Sources

Despite the relative consistency of the results, our survey protocol has some limitations. Herd positions are only approximated by the GPS fixes. Sensors can be more or less precise, depending on signal quality, and the individual animal wearing the collar may not always be within the herd and certainly not at its centroid at all times. Recent studies [32] show that optimal precision can be achieved by equipping a leading individual or, ideally, the one with the highest level of social connectivity in the herd. However, the high level of precision reached for each individual animal equipped can be of great interest for reusing the data in behavioural studies. Additionally, idiosyncrasies may arise from the relatively small number of herds that we followed, as Figure 6 illustrates. However, we consider that the amount of data we collected mitigates these imprecisions in the results of this paper. Additionally, the accuracy of the sensors is 95% within 10 m, which is far below the differences in values between the parameters of the statistical laws used by the HMM to discriminate between states.

The frequency of one observation every 30 min was chosen because we felt it was sufficient to allow the identification of a grazing state by the model, while allowing a 2-year-long survey, which covered two full transhumance journeys. Obviously, some features of real herds’ behaviour occur in time periods lower than 30 min. However, we believe that this allowed us to obtain plausible behaviour states, in particular given the approximation that the GPS location of a single animal represents its entire herd. Additionally, such a frequency fitted satisfactorily with the spatial resolution of the geographical data given the speeds at which herds movements occurred.

The combination of the dilution of precision of the GPS sensors and the spatial resolution of the satellite rasters used to generate results on the nature of the landscape where herd activity occurred presents another significant limitation to our results. This is particularly true for the Globcover-based dataset used outside the home-village area. Additionally, automatic and semi-automatic spatial land-unit attribution has a limited level of confidence, and false positives may have occurred. More recent and precise products would prove valuable in interpreting our data; however, quality geographical data on West Africa are difficult to obtain.

### 5.2. Model Fit, Validity and Means of Improvement

The quality of states prediction by a hidden Markov model is directly illustrated by its likelihood and, therefore, its BIC. Discussing the validity of the model would thus typically involve comparing fit metrics with similar experiments from the literature. However, the methodology of fitting a hidden Markov model to GPS tracking data mostly belongs to the field of movement ecology (e.g., [33]). To our knowledge, no past reference exists on the use of hidden Markov models on domestic cattle in arid environments. Nevertheless, it is possible to compare the model we found with what has been conducted on similar wild animals. It is coherent with what Beumer et al. [34] found on wild large *Bovidae* in an arid but cold environment—they describe a three-state model with comparable metrics. Franke et al. [35] had comparable metrics on *Cervidae* as well.

Another form of validation is to check for the practical plausibility of the results. Speeds in the foraging state (Section 3.1) appear slower than the standard walking speeds of domestic cattle (e.g., [36]). However, the direct path between two GPS positions is unlikely to correspond to the actual movement of a herd or animal, so this appears reasonable. Indeed, total distances travelled per day (Table 3) by transhumant herds are consistent with the findings of Turner and Schlecht [37] and Zampaligré and Schlecht [38] elsewhere in the continent. Resident herds travel significantly shorter average distances in comparison, which is likely the consequence of more sedentary herding practices.

Care should be taken when extrapolating the activity states from an unsupervised classification algorithm such as an HMM to real herd behaviour. Direct observation of herds would provide a more reliable interpretation of which activity they perform when producing the different types of trajectories considered by the model. Some high-frequency monitoring with direct behaviour assessment, for example, with the use of a decision tree [26], may have been a way to generate a calibration dataset. Additional improvements to the quality of the model may arise from the addition of variables, aside from the trajectory metrics, such as acceleration or orientation [39,40]. Data gaps, whether related to data collection or preparation, mentioned in Section 2.3, are a source of bias in the accuracy of the model. We made the decision to leave out entire parts of the dataset to avoid gaps. However, there are workarounds for this problem, either by temporal interpolation for short gaps (e.g., [27]) or by separating a sequence of observations into two or more “virtual animals” when the data allow for larger gaps.

### 5.3. Herd Behaviour

Some characteristics of herd behaviour that we highlight are consistent with the literature and field observations. The mid-day rest peak, identified in Section 4.1, is observed in the field. Observations being labelled in the resting state at night (Section 4.1) are consistent with the resident herds being tethered. On the other hand, our results show nocturnal foraging for transhumant herds (Section 4.1). It is not a surprising behaviour, as it is less energetically costly to graze at colder periods and is common for ungulates [41] and consistent with observations reported by Chirat et al. [42] on nomadic cattle from the pastoral area of Senegal. Yet, it is the first time, to our knowledge, that this phenomenon is quantified in such a way.

The variation of state transition probability with temperature measured by the collar (Section 3.1) as a covariate appears to be the result of an effect of environmental temperature on herd behaviour. It could be an indirect effect of the environmental temperature shift between seasons. However, seasonal variations in recorded temperatures are relatively small (see S1), which seems to invalidate this hypothesis. Thus, we believe this could mask an effect of the time of day. Low temperatures correspond to nighttime, where the resting state should be more stable, in particular for tethered herds. This would be an explanation of the observations reported at the end of Section 3.1.

The comparison of the behaviour model with those generated from fits on subsets for transhumant and resident herds (Section 3.3) shows that it is highly representative of the movements of transhumant agropastoral herds. In this paper, however, training our HMM on the entire dataset ensures that the travelling state is isolated from the foraging state, even for resident herds (as discussed Section 3.2). In conclusion, the model we present here can be used on similar datasets with little concern, particularly if the data are only collected on transhumant agropastoral cattle. It provides information that might not be available when working solely with non-transhumant (resident) cattle.

### 5.4. Herding Behaviour and Mobility Patterns in the Transhumance Journey

Individual fallowing, Section 4.3, has not been documented to our knowledge. Odru [14], however, suggested that parcels of land are rarely left uncultivated in our study area, and only by accident. However, her work was conducted in a village with collective fallow, so individual fallowing might only happen in villages without collective fallow.

As highlighted in Section 4.3, resident herds spend a substantial portion of their nights in homefields during the dry seasons, notably less than the time spent in homefields during the daytime. This pattern suggests the occurrence of fertility transfer, which was repeatedly identified in these systems [2,43].

The route of the transhumant herds is based on several factors yet mainly relies on the social network between farmers and relatives who migrated east at the end of the 1970s [44]. The longest stops we describe occur mostly on land that belongs to these relatives. We believe there may be an opportunity to discuss the benefits of contract corralling [45], in which our surveyed herds participate (see Section 4.3).

Dates of departure and return are consistent with what Faye et al. [13] described two decades ago. Further work on a larger set of herds appears necessary to confirm that land-use change and global changes have seemingly not affected Senegalese transhumance over that duration.

### 5.5. A Methodology to Produce Original Insights on Pastoral Practices

HMMs are widely used to analyse animal mobility in ecological research, notably in studies of wild ungulates, as stated above [34,35]. They have been applied, albeit marginally, on non-pastoral livestock farming [46]. Yet, to our knowledge, this study represents the first application of HMMs to pastoral livestock systems on such large temporal and geographical scales.

Our thematic results are mostly in line with current knowledge of herding practices in the former groundnut basin of Senegal. In particular, daytime foraging and browsing in rangelands and croplands—mostly bushfields—and nighttime corralling on homefields are clearly shown. However, some results highlight aspects which are little known (nocturnal foraging, Section 4.1; temporal quantification of transhumant phases, Section 4.2) or are original findings (individual fallowing, Section 4.3). Therefore, we believe this is an indication of the great value that such methodology can have.

Additionally, the tools we have developed in the HAMAC routine and the *moveHMM* package bring to the table tools, which have strong potential for more in-depth pastoral studies in the Sahel.

We have given an example in Figure 3 of how it is possible to calculate the effect of the temperature recorded by the collars on the behavioural model. The ability to estimate the effect of covariates on behaviour can prove very useful in exploring the effect of other environmental factors, such as distance to the paddock or to the drinking point, rainfall or vegetation data, for example. Beumer et al. [34] provide some examples on wild fauna. Although HMMs and the *moveHMM* package offer valuable tools for integrating covariates to a model, alternative platforms are used in ecology, such as the *momentuHMM* package by the same development team [47] or *amt* [48], which is based on step-selection functions.

Training models on data subsets after a survey, as we did in Section 3.3, has the potential to address thematic questions and strengthen explanatory or predictive models [27]. In particular, subsets for the transhumance phases that we highlighted in Section 4.2 can show an interesting evolution of herding practices, and therefore resource use, throughout the journey.

The ability to simulate virtual mobility (as shown in Table 1) can be essential for studies on herding decision-making (e.g., [49]). The data collected also have good potential when used to validate the results of such models [50].

It should be noted, however, that fitting an HMM on our dataset proved relatively costly in terms of computing power. It could thus be more accessible, if conducting a similar survey, to directly reuse the fitted model on the novel dataset. Indeed, we showed in Section 3.3 that the model on which we rely in this paper, presented in Section 3.1, can be easily used on GPS surveys of cattle in the Sahel with a minimum amount of modifications. Additionally, the entire dataset of GPS locations and most likely activity state will be made available via a planned data paper.

### 5.6. GPS Surveys and HMM for Sahel Pastoralism: Some Prospects

A few paths open for the pastoralist who seeks to map herd activities using GPS tracking and HMMs.

Pastoral mobility is obviously partially driven by what we might call the natural behaviour of animals, which is quite well documented [51]. However, this behaviour is mainly constrained by the choice of herders to avoid certain areas, select certain paths and destinations, and release, tether or corral animals at certain points of the day. This choice depends on several factors [52] but above all on the availability of resources [37,42]. Combining our GPS survey data and land-use data proved fruitful (Section 4.3), but more sophisticated data on available biomass, both quantitative and qualitative, as proposed in other examples [53,54,55], could generate benefits by discussing these factors. Indeed, the literature [38,42] suggests that the availability of forage restricts mobility and therefore its local intensity of use. Additionally, combining grazing locations and vegetation data would provide insights on resource use, and such a study could be expanded to manure depots to further discuss fertility transfers, as proposed by Auerswald et al. [56] and modelled by Chirat [57].

A good understanding of herds behaviour dynamics identified in Section 4.1 may benefit from adopting an energetic approach (e.g., [41,58]). For example, greater floristic diversity appears with the first rains, and with it comes selection behaviour, which may induce longer foraging periods. However, at the same time, the availability of plant biomass increases, which should reduce foraging time. However, the impact of human herding management, discussed above, implies that the most crucial point is to understand its socio-technical conditions through surveys.

Although the present work, and in particular Section 4.2, has shed some light on transhumance journeys in Senegal, it may prove of interest to further process results in quantitative approaches [59,60] to discuss the activity in its entirety.

Although the agropastoral farmers of the villages of origin in the study area only send cattle for transhumance, pastoral and (semi-)nomadic farmers across the Sahel move in multi-species droves. It may be interesting to expand the sample of species in subsequent studies. In such a case, it may be desirable to repeat the approach presented in Section 3.2 to select the adequate number of states to identify behavioural features of interest. This approach could help address questions regarding the use of pastoral resources, as did Zampaligré and Schlecht [38], and can also provide valuable information on inter- and cross-species transmission of diseases, an area that currently lacks sufficient mobility surveillance data [61].

In this paper, we have opted for a very simplistic typology of two types of herd. Blanchard et al. [62] show that deeper analysis of the dataset with land use has the potential to further discriminate herds and establish functional typologies of herds and herding practices.

## 6. Conclusions

The objective of this study was to propose a methodology to make the most of a GPS survey of pastoral herds. We collected a large-scale, high-frequency dataset of GPS locations of transhumant and resident cattle herds in agropastoral Senegal, which proved fruitful in identifying key and undocumented features of pastoral mobility in agro-sylvo-pastoral systems in the Sahel. We developed an original behaviour model using the hidden Markov framework, typically used in ecological research, to create insights on agropastoral livestock practices. We managed to robustly fit an optimal three-state model to the collected data. This model predicts changes between states of mobility, and we showed how the probability of these changes vary with temperature, which we believe to be an effect of the time of the day. We displayed how these states are distributed over time during the day and throughout the seasons, highlighting the poorly documented behaviour of nocturnal foraging for Sahel cattle. We showed how transhumance is a phasic journey and computed precise metrics of these phases. We combined the data with landscape use to show in which environment the herds perform their activities, highlighting, in particular, that individual fallowing may occur.

We believe that the behaviour model we present in this paper may be built on in the future with high added value. It is to that end that we detailed our process of finding out the optimal three states to address our research question and how we found that the use of sub datasets for resident and transhumant herds was superfluous. It can be used on lower frequency, easier-to-collect or larger scale datasets to identify foraging periods or location, thus improving knowledge of the factors that influence herd behaviour and pastoral mobility on a large scale. It offers the potential for novel insights by assessing the influence of environmental factors on herding practices through the use of covariates. Additionally, the ability to simulate virtual animals can be used in predictive approaches, which may be of use in addressing disease transmission. The parameters presented in this paper can be adapted directly on a similar dataset, and the entire R script for data processing and parametrisation is available at https://gitlab.cirad.fr/selmet/hamac (accessed on 15 October 2024) [7]. It could be used on different species or applied to other pastoral systems in the Sahel. In addition, the dataset resulting from this study is available in open access (at [6]). Our findings offer a preliminary glimpse into its possible use in African pastoralist studies, which will require surveys of farmers.

## Figures and Tables

**Table 1:** Parameters of the distribution laws of the probability of emission of observed trajectory metrics for each state of the optimal behavioural model. For each state, left (Samp. lim.) are the lower and upper limits of the uniform distribution used for sampling initial conditions, right are the resulting parameters of the optimal model (bold) and the lower and upper boundaries of their confidence intervals. Below are simulated trajectories on 10 points for each state. μ: mean step length, σ: step length standard deviation, $\mu^{\prime }$: mean angle, κ: angle concentration, ζ: zero-inflation (see Michelot et al. [21]), $p_{0}$: proportion of observations of the dataset where step length is equal to zero km, equal to $1.93771\times 10^{-4}$, NA: no data.

	Resting State		Foraging State		Travelling State	
	Samp. lim.	Result (±CI 95%)	Samp. lim.	Result (±CI 95%)	Samp. lim.	Result (±CI 95%)
μ (m/30 min)	5–100	**13.53** (13.46–13.61)	50–250	**180.1** (178.8–181.5)	100–1000	**685.4** (680.6–690.4)
σ (m/30 min)	5–100	**11.99** (11.91–12.08)	50–500	**149.2** (148.0–150.5)	100–1000	**439.8** (436.9–442.9)
μ’ (rad/30 min)	−3–3	**−3.05** (−3.07–3.03)	−3–3	**0.00** (−0.0360–0.0361)	−0.5–0.5	**−0.0146** (−0.0211–0.00822)
κ (rad/30 min)	0.1–1	**0.29** (0.28–0.30)	1.5–5	**0.19** (0.18–0.20)	1–15	**1.95** (1.93–1.98)
ζ (dimensionless)	$p_{0}$	**$4.2\times 10^{-4}$** ($3.4\times 10^{-4}$–$5.1\times 10^{-4}$)	$p_{0}/100$	**$9.5\times 10^{-13}$** (NA–NA)	$p_{0}/100$	**$7.13\times 10^{-12}$** ($7.13\times 10^{-12}$–$7.13\times 10^{-12}$)
Simulated trajectories on 10 points	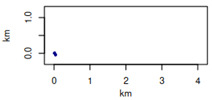		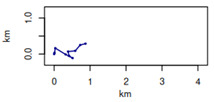		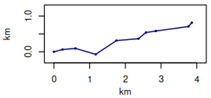	

**Table 2:** Trajectory parameters for optimal models fitted with two to five activity states. μ, mean of step length, σ, standard deviation of step length, $\mu^{\prime }$, mean of angle, κ, angle concentration, ζ, zero inflation. The second number in parentheses is the number of ran fits; the first is how many settled on the optimal model. BIC, Bayesian information criterion; lower means a better fit. Selected model highlighted in grey.

	St.	μ	σ	μ’	κ	ζ	Interpretation
		m·30 min^−1^	m·30 min^−1^	rad·30 min^−1^	rad·30 min^−1^	*dimension less*	
Two-st. m. (23/44)	1	16	14	−3.06	0.32	$4.0\times 10^{-4}$	Resting
BIC: 488 768	2	351	350	−0.01	0.65	$4.0\times 10^{-11}$	Moving
Three-state model	1	13	12	−3.05	0.29	$4.2\times 10^{-4}$	Resting
(44/110)	2	180	149	0.00	0.19	$9.5\times 10^{-13}$	Foraging
BIC: 394 277	3	685	440	−0.01	1.95	$1.5\times 10^{-10}$	Travelling
Four-state model (6/22) BIC: 337 142	1	7	5	−3.09	0.23	$7.4\times 10^{-4}$	Resting
	2	26	19	−3.03	0.38	$1.8\times 10^{-10}$	Active resting
	3	203	174	0.03	0.22	$3.7\times 10^{-6}$	Foraging
	4	663	458	−0.02	2.11	$5.6\times 10^{-13}$	Travelling
	1	6	4	−3.09	0.23	$8.4\times 10^{-4}$	Resting
Five-state model	2	23	16	−3.02	0.34	$7.8\times 10^{-24}$	Active resting
(21/44)	3	144	109	0.08	0.06	$2.4\times 10^{-42}$	Foraging
BIC: 333 786	4	472	301	−0.02	1.24	$4.6\times 10^{-51}$	Short travel
	5	1421	349	0.00	7.98	$3.3\times 10^{-11}$	Long travel

**Table 3:** Average daily distance covered and time spent in each state for resident and transhumant herds during daytime exclusively. *d*: distance, *t*: time.

	Resting State		Foraging State		Travelling State	
	*d* (m/day)	*t* (h/day)	*d* (m/day)	*t* (h/day)	*d* (m/day)	*t* (h/day)
Resident	116	14.9	2330	6.94	3400	2.22
Transhu.	53.2	9.57	2570	8.93	6330	4.70

## Data Availability

The Herds Activity Mapping and Analytical Classification (HAMAC) routine is available at https://gitlab.cirad.fr/selmet/hamac (accessed on 15 October 2024). The dataset of GPS positions, resulting behaviour and corresponding land-use presented in this article are accessible online, under the title: “Mobility, Behaviour and Land-Use Data from GPS Tracking and Hidden Markov Model Training on Cattle Herds in Sahel Agropastoral Systems“, at https://doi.org/10.18167/DVN1/GHJKQO (accessed on 15 October 2024).

## Supplementary Materials

The following supporting information can be downloaded at: https://www.mdpi.com/article/10.3390/s24216964/s1, Figure S1: Distribution of the temperatures measured by the collars with each GPS location over the survey during daytime and nighttime and for each type of heard.; Figure S2: Illustration of the superposition of trees over several land units: land-use interpretation over part of the 1.5 m resolution SPOT7 image.
