# Supplementary figures and images for: GPS-Based Hidden Markov Models to Document Pastoral Mobility in the Sahel
Source: Sensors (Basel). 2024 Oct 30;24(21):6964. doi: 10.3390/s24216964 (PMC11548461; doi:10.3390/s24216964)

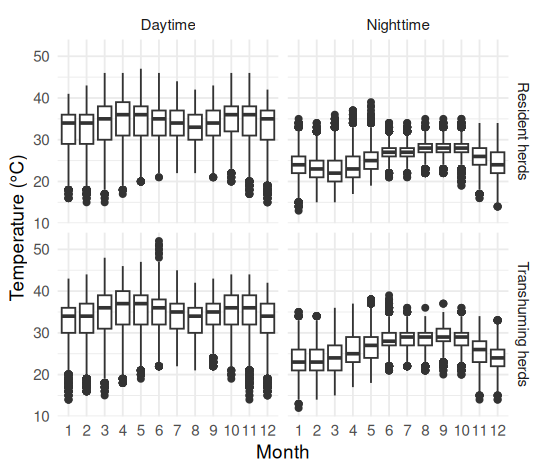

Supplement: Supplementary file 1 [file sensors-24-06964-s001.zip › S1-240510-TMPbox.png]

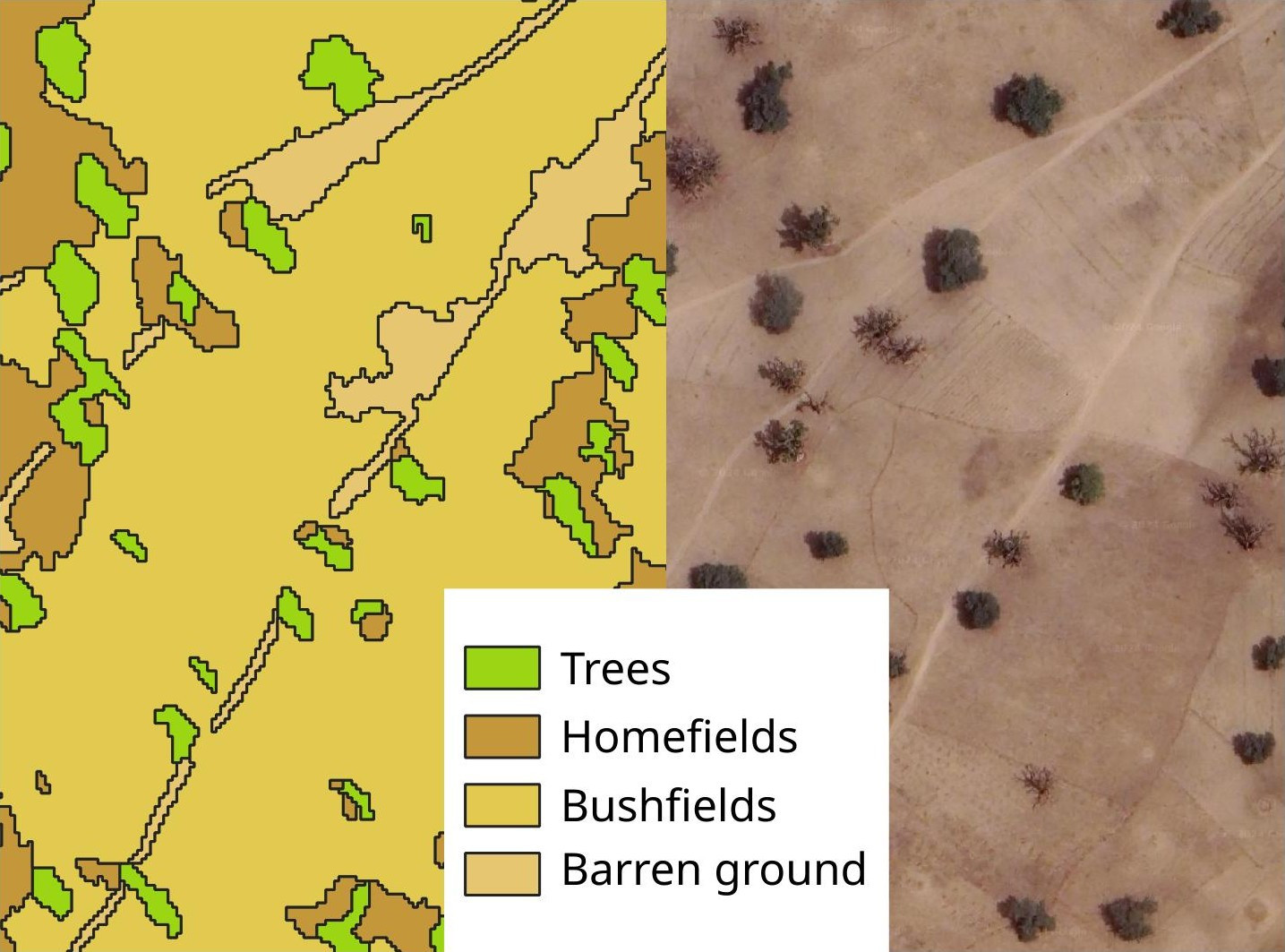

Supplement: Supplementary file 1 [file sensors-24-06964-s001.zip › S2-IllusTrees.jpeg]
